# 2,2,2-Trichloro-*N*-(3-nitro­phen­yl)acetamide

**DOI:** 10.1107/S1600536812036732

**Published:** 2012-09-01

**Authors:** A. N. Prabhu, V. Upadhyaya, K. S. Girisha, C. S. Naveena, T. N. Guru Row

**Affiliations:** a Physics Department, Manipal Institute of Technology, Manipal University, Manipal 576 104, India; b Solid State and Sructural Chemistry Unit, Indian Institute of Science, Bangalore 560 012, India; c Department of Chemistry, Mangalore University 575 199, Karnataka, India

## Abstract

In the title compound, C_8_H_5_Cl_3_N_2_O_3_, the dihedral angle between the nitro­phenyl ring and the acetamide group is 5.47 (6)°. In the crystal, N—H⋯O and C—H⋯O hydrogen bonds link the mol­ecules into chains running parallel to the *b* axis.

## Related literature

For background to acetamides, see: Khan *et al.* (2010 ▶); Tahir & Shad (2011 ▶). For a related structure, see: Rosli *et al.* (2007 ▶). For hydrogen-bond motifs, see: Bernstein *et al.* (1995 ▶).
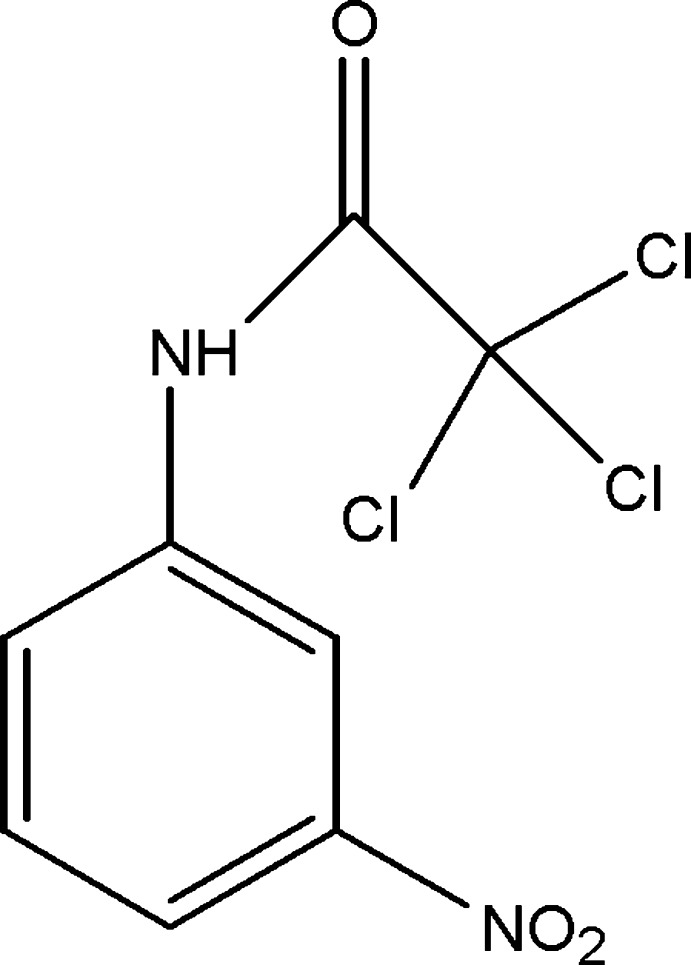


## Experimental

### 

#### Crystal data

C_8_H_5_Cl_3_N_2_O_3_
*M* *_r_* = 283.49
Orthorhombic, 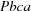

*a* = 11.5164 (8) Å
*b* = 10.1427 (5) Å
*c* = 19.9054 (11) Å
*V* = 2325.1 (2) Å^3^
*Z* = 8
Mo *K*α radiation
μ = 0.78 mm^−1^
*T* = 296 K
0.20 × 0.18 × 0.18 mm

#### Data collection

Bruker SMART APEX CCD detector diffractometer
Absorption correction: multi-scan (*SAINT-Plus*; Bruker, 1998 ▶) *T*_min_ = 0.860, *T*_max_ = 0.872
8739 measured reflections
2532 independent reflections
1713 reflections with *I* > 2σ(*I*)
*R*_int_ = 0.028

#### Refinement

*R*[*F* ^2^ > 2σ(*F* ^2^)] = 0.049
*wR*(*F* ^2^) = 0.144
*S* = 1.07
2532 reflections
145 parameters
H-atom parameters constrained
Δρ_max_ = 0.46 e Å^−3^
Δρ_min_ = −0.37 e Å^−3^

### 

Data collection: *SMART* (Bruker, 1998 ▶); cell refinement: *SAINT-Plus* (Bruker, 1998 ▶) (Bruker, 1998 ▶); data reduction: *SAINT-Plus*; program(s) used to solve structure: *SHELXS97* (Sheldrick, 2008 ▶); program(s) used to refine structure: *SHELXL97* (Sheldrick, 2008 ▶); molecular graphics: *ORTEP-3* (Farrugia, 1997 ▶) and *CAMERON* (Watkin *et al.*, 1993 ▶); software used to prepare material for publication: *WinGX* (Farrugia, 1999 ▶).

## Supplementary Material

Crystal structure: contains datablock(s) global, I. DOI: 10.1107/S1600536812036732/pv2576sup1.cif

Structure factors: contains datablock(s) I. DOI: 10.1107/S1600536812036732/pv2576Isup2.hkl

Supplementary material file. DOI: 10.1107/S1600536812036732/pv2576Isup3.cml

Additional supplementary materials:  crystallographic information; 3D view; checkCIF report

## Figures and Tables

**Table 1:** Hydrogen-bond geometry (Å, °)

*D*—H⋯*A*	*D*—H	H⋯*A*	*D*⋯*A*	*D*—H⋯*A*
C4—H4⋯O1^i^	0.93	2.59	3.345 (4)	138
N2—H2*N*⋯O1^i^	0.86	2.15	2.990 (3)	164

Symmetry code: (i) 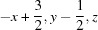.

**Fig. 1.**
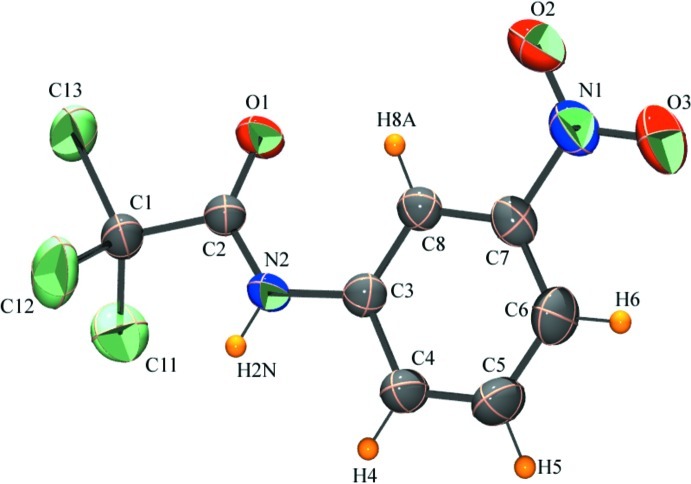
ORTEP (Farrugia, 1997) view of the title compound, showing 50% probability ellipsoids and the atom numbering scheme.

**Fig. 2.**
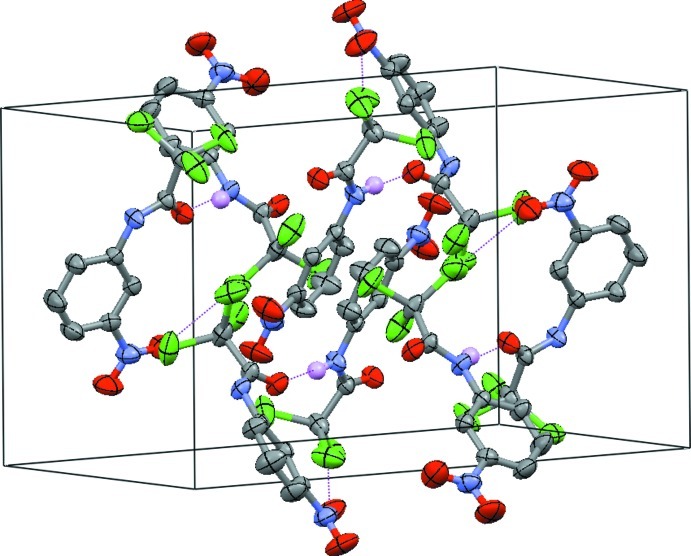
A unit cell packing of the title compound showing intermolecular interactions with dotted lines. H-atoms not involved in hydrogen bonding have been excluded.

## supplementary crystallographic information

### Comment

In view of earlier studies and interest owing to the biological activity of acetamide (Khan *et al.*, 2010; Tahir & Shad, 2011), we report herein the crystal structure of the title compound. In the title compound, the nitro phenyl ring makes a dihedral angle of 5.47 (6)° with acetamide group. Bond lengths and angles are within the normal ranges and are comparable with a related structure (Rosli *et al.*, 2007). In the crystal, an S(6) ring motif (Bernstein *et al.*, 1995) is formed *via* intramolecular C8—H8A···O1 hydrogen bond (Table 1). The C—H···O and C—H···N hydrogen bonding intractions (Table 1 and Figure 2) result in bifurcated bonds and link the molecules into chains along the *b*-axis.

### Experimental

3-Nitroaniline (1.0 g, 0.0072 mmol) was dissolved in 2*M* hydrochloric acid (5.0 ml), and added a little crushed ice. A solution of hydrated sodium acetate (5.0 g) in 25 ml of water was introduced, followed by trichloroacetic anhydride (4.5 g, 0.01457 mol). The mixture was shaken in the cold until the smell of trichloroacetic anhydride disappeared. The title compound was collected by filtration and recrystallized from an aqueous ethanol (75%) solution; yield: 1.52 g (75%), m.p. 376.15–378.15 K.

### Refinement

The H atoms were placed at calculated positions in the riding model approximation with N—H = 0.86 and C—H = 0.93 Å, and *U*_iso_(H) = 1.2*U*_eq_(N/C).

### Crystal data

**Table:**

C_8_H_5_Cl_3_N_2_O_3_	*F*(000) = 1136
*M**_r_* = 283.49	*D*_x_ = 1.620 Mg m^−^^3^
Orthorhombic, *P**b**c**a*	Mo *K*α radiation, λ = 0.71073 Å
Hall symbol: -P 2ac 2ab	Cell parameters from 2532 reflections
*a* = 11.5164 (8) Å	θ = 2.1–27.0°
*b* = 10.1427 (5) Å	µ = 0.78 mm^−^^1^
*c* = 19.9054 (11) Å	*T* = 296 K
*V* = 2325.1 (2) Å^3^	Block, yellow
*Z* = 8	0.20 × 0.18 × 0.18 mm

### Data collection

**Table:**

Bruker SMART APEX CCD detector diffractometer	2532 independent reflections
Radiation source: Enhance (Mo) X-ray Source	1713 reflections with *I* > 2σ(*I*)
Graphite monochromator	*R*_int_ = 0.028
ω scans	θ_max_ = 27.0°, θ_min_ = 2.1°
Absorption correction: multi-scan (*SAINT-Plus*; Bruker, 1998)	*h* = −5→14
*T*_min_ = 0.860, *T*_max_ = 0.872	*k* = −10→12
8739 measured reflections	*l* = −14→25

### Refinement

**Table:**

Refinement on *F*^2^	Primary atom site location: structure-invariant direct methods
Least-squares matrix: full	Secondary atom site location: difference Fourier map
*R*[*F*^2^ > 2σ(*F*^2^)] = 0.049	Hydrogen site location: inferred from neighbouring sites
*wR*(*F*^2^) = 0.144	H-atom parameters constrained
*S* = 1.07	*w* = 1/[σ^2^(*F*_o_^2^) + (0.0695*P*)^2^ + 0.9222*P*] where *P* = (*F*_o_^2^ + 2*F*_c_^2^)/3
2532 reflections	(Δ/σ)_max_ = 0.001
145 parameters	Δρ_max_ = 0.46 e Å^−^^3^
0 restraints	Δρ_min_ = −0.37 e Å^−^^3^

### Special details

**Table:**

Geometry. All s.u.'s (except the s.u. in the dihedral angle between two l.s. planes) are estimated using the full covariance matrix. The cell s.u.'s are taken into account individually in the estimation of s.u.'s in distances, angles and torsion angles; correlations between s.u.'s in cell parameters are only used when they are defined by crystal symmetry. An approximate (isotropic) treatment of cell s.u.'s is used for estimating s.u.'s involving l.s. planes.
Refinement. Refinement of *F*^2^ against ALL reflections. The weighted *R*-factor *wR* and goodness of fit *S* are based on *F*^2^, conventional *R*-factors *R* are based on *F*, with *F* set to zero for negative *F*^2^. The threshold expression of *F*^2^ > 2σ(*F*^2^) is used only for calculating *R*-factors(gt) *etc*. and is not relevant to the choice of reflections for refinement. *R*-factors based on *F*^2^ are statistically about twice as large as those based on *F*, and *R*- factors based on ALL data will be even larger.

### Fractional atomic coordinates and isotropic or equivalent isotropic displacement parameters (Å^2^)

**Table:**

	*x*	*y*	*z*	*U*_iso_*/*U*_eq_	
C1	0.8599 (3)	0.8973 (3)	0.29007 (15)	0.0522 (8)	
C2	0.7513 (2)	0.9294 (2)	0.33324 (13)	0.0390 (6)	
C3	0.5939 (2)	0.8197 (2)	0.39617 (13)	0.0367 (6)	
C4	0.5685 (3)	0.7018 (3)	0.42914 (16)	0.0520 (7)	
H4	0.6196	0.6310	0.4260	0.062*	
C5	0.4687 (3)	0.6897 (3)	0.4662 (2)	0.0705 (10)	
H5	0.4522	0.6103	0.4876	0.085*	
C6	0.3927 (3)	0.7935 (3)	0.47228 (18)	0.0621 (9)	
H6	0.3243	0.7852	0.4968	0.074*	
C7	0.4209 (2)	0.9099 (3)	0.44096 (14)	0.0429 (6)	
C8	0.5197 (2)	0.9263 (2)	0.40254 (13)	0.0387 (6)	
H8A	0.5358	1.0063	0.3817	0.046*	
Cl1	0.82157 (11)	0.79451 (9)	0.22201 (5)	0.0843 (4)	
Cl3	0.92258 (9)	1.04369 (8)	0.25994 (5)	0.0801 (4)	
Cl2	0.96357 (8)	0.81360 (10)	0.34058 (6)	0.0828 (3)	
N1	0.3404 (2)	1.0223 (3)	0.44728 (15)	0.0580 (7)	
N2	0.6956 (2)	0.82155 (19)	0.35614 (12)	0.0433 (6)	
H2N	0.7247	0.7464	0.3454	0.052*	
O1	0.72365 (18)	1.04229 (16)	0.34446 (11)	0.0524 (5)	
O2	0.3623 (2)	1.1225 (2)	0.41711 (16)	0.0858 (8)	
O3	0.2570 (2)	1.0101 (3)	0.48485 (16)	0.0900 (9)	

### Atomic displacement parameters (Å^2^)

**Table:**

	*U*^11^	*U*^22^	*U*^33^	*U*^12^	*U*^13^	*U*^23^
C1	0.0626 (19)	0.0350 (14)	0.0589 (18)	−0.0020 (13)	0.0223 (15)	0.0010 (12)
C2	0.0444 (14)	0.0307 (12)	0.0418 (14)	−0.0010 (11)	0.0055 (13)	0.0007 (10)
C3	0.0383 (14)	0.0318 (12)	0.0400 (13)	−0.0053 (10)	−0.0002 (12)	−0.0019 (10)
C4	0.0499 (17)	0.0353 (14)	0.071 (2)	0.0007 (12)	0.0134 (16)	0.0083 (13)
C5	0.064 (2)	0.0461 (18)	0.101 (3)	−0.0008 (15)	0.027 (2)	0.0226 (17)
C6	0.0453 (17)	0.0588 (19)	0.082 (2)	−0.0057 (14)	0.0193 (17)	0.0078 (16)
C7	0.0368 (14)	0.0433 (14)	0.0485 (15)	0.0011 (11)	−0.0027 (13)	−0.0063 (12)
C8	0.0412 (14)	0.0332 (13)	0.0416 (14)	−0.0015 (10)	−0.0005 (12)	−0.0017 (10)
Cl1	0.1194 (9)	0.0689 (6)	0.0645 (6)	−0.0233 (5)	0.0383 (6)	−0.0211 (4)
Cl3	0.0991 (8)	0.0439 (4)	0.0973 (7)	−0.0162 (4)	0.0522 (6)	0.0022 (4)
Cl2	0.0527 (5)	0.0816 (6)	0.1141 (8)	0.0127 (4)	0.0146 (5)	0.0168 (6)
N1	0.0462 (15)	0.0550 (16)	0.0728 (18)	0.0068 (12)	0.0062 (14)	−0.0033 (13)
N2	0.0482 (13)	0.0234 (10)	0.0584 (14)	0.0020 (9)	0.0148 (11)	0.0000 (9)
O1	0.0560 (12)	0.0243 (9)	0.0767 (14)	−0.0014 (8)	0.0169 (11)	−0.0020 (8)
O2	0.0786 (18)	0.0568 (15)	0.122 (2)	0.0245 (13)	0.0300 (16)	0.0152 (15)
O3	0.0544 (15)	0.0901 (18)	0.125 (2)	0.0202 (13)	0.0345 (16)	0.0105 (17)

### Geometric parameters (Å, º)

**Table:**

C1—C2	1.552 (4)	C5—C6	1.374 (4)
C1—Cl3	1.756 (3)	C5—H5	0.9300
C1—Cl1	1.766 (3)	C6—C7	1.374 (4)
C1—Cl2	1.777 (3)	C6—H6	0.9300
C2—O1	1.209 (3)	C7—C8	1.380 (4)
C2—N2	1.348 (3)	C7—N1	1.476 (4)
C3—C8	1.385 (3)	C8—H8A	0.9300
C3—C4	1.395 (3)	N1—O2	1.207 (3)
C3—N2	1.417 (3)	N1—O3	1.223 (4)
C4—C5	1.372 (4)	N2—H2N	0.8600
C4—H4	0.9300		
			
C2—C1—Cl3	110.05 (18)	C6—C5—H5	119.6
C2—C1—Cl1	110.3 (2)	C7—C6—C5	117.9 (3)
Cl3—C1—Cl1	109.88 (16)	C7—C6—H6	121.1
C2—C1—Cl2	109.17 (19)	C5—C6—H6	121.1
Cl3—C1—Cl2	108.75 (18)	C6—C7—C8	123.4 (3)
Cl1—C1—Cl2	108.65 (15)	C6—C7—N1	118.5 (3)
O1—C2—N2	125.5 (2)	C8—C7—N1	118.1 (2)
O1—C2—C1	120.9 (2)	C7—C8—C3	117.7 (2)
N2—C2—C1	113.6 (2)	C7—C8—H8A	121.1
C8—C3—C4	119.8 (2)	C3—C8—H8A	121.1
C8—C3—N2	123.5 (2)	O2—N1—O3	123.6 (3)
C4—C3—N2	116.7 (2)	O2—N1—C7	118.5 (3)
C5—C4—C3	120.4 (3)	O3—N1—C7	117.8 (3)
C5—C4—H4	119.8	C2—N2—C3	126.5 (2)
C3—C4—H4	119.8	C2—N2—H2N	116.8
C4—C5—C6	120.8 (3)	C3—N2—H2N	116.8
C4—C5—H5	119.6		
			
Cl3—C1—C2—O1	−3.2 (4)	C6—C7—C8—C3	−0.6 (4)
Cl1—C1—C2—O1	−124.6 (3)	N1—C7—C8—C3	−179.1 (2)
Cl2—C1—C2—O1	116.1 (3)	C4—C3—C8—C7	−1.2 (4)
Cl3—C1—C2—N2	177.6 (2)	N2—C3—C8—C7	177.4 (2)
Cl1—C1—C2—N2	56.2 (3)	C6—C7—N1—O2	−176.5 (3)
Cl2—C1—C2—N2	−63.1 (3)	C8—C7—N1—O2	2.1 (4)
C8—C3—C4—C5	1.9 (5)	C6—C7—N1—O3	6.1 (4)
N2—C3—C4—C5	−176.8 (3)	C8—C7—N1—O3	−175.3 (3)
C3—C4—C5—C6	−0.8 (6)	O1—C2—N2—C3	1.3 (5)
C4—C5—C6—C7	−1.0 (6)	C1—C2—N2—C3	−179.5 (3)
C5—C6—C7—C8	1.8 (5)	C8—C3—N2—C2	18.2 (4)
C5—C6—C7—N1	−179.7 (3)	C4—C3—N2—C2	−163.2 (3)

### Hydrogen-bond geometry (Å, º)

**Table:**

*D*—H···*A*	*D*—H	H···*A*	*D*···*A*	*D*—H···*A*
C8—H8*A*···O1	0.93	2.32	2.870 (3)	117
C4—H4···O1^i^	0.93	2.59	3.345 (4)	138
N2—H2*N*···O1^i^	0.86	2.15	2.990 (3)	164

Symmetry code: (i) −*x*+3/2, *y*−1/2, *z*.
